# Validation of the 2011 and 2016 American college of rheumatology diagnostic criteria for fibromyalgia in a Chinese population

**DOI:** 10.1080/07853890.2023.2249921

**Published:** 2023-08-26

**Authors:** Juan Jiao, Zeng-yu Cheng, Yu-ya Xiao, Hui Wang, Yong-feng Zhang, Ya-yun Zhao, Yuan Jia

**Affiliations:** a Rheumatology Department, Guang’anmen Hospital, China Academy of Chinese Medical Sciences, Beijing, China; b Rheumatology Department, Shenzhen Traditional Chinese Medicine Hospital, Shenzhen, China; c Department of Rheumatology, The First Affiliated Hospital of Harbin Medical University, Harbin, China; d Department of Rheumatology and Clinical Immunology, Beijing Chao Yang Hospital, Capital Medical University, Beijing, China; e Rheumatology Department, Hebei Hospital of Traditional Chinese Medicine, Shijiazhuang, China; f Department of Rheumatology and Immunology, Peking University People’s Hospital & Beijing Key Laboratory for Rheumatism Mechanism and Immune Diagnosis, Beijing, China

## Abstract

**Purpose:**

To provide a foundation for clinical diagnosis, epidemiological investigation and intervention trials, we examined the reliability and validity of the American College of Rheumatology (ACR) 2011 and 2016 survey diagnostic criteria among Chinese patients based on the fibromyalgia severity (FS) scale.

**Methods:**

In this study, 200 fibromyalgia patients diagnosed according to the 1990 criteria (1990c) were matched with rheumatoid arthritis (RA) patients based on age and gender. The FS scale score and its subscales were examined to determine their correlations with the revised fibromyalgia impact questionnaire (FIQR). Receiver operator characteristic (ROC) analysis was performed, and test-retest reliability, internal consistency, and construct validity were examined.

**Results:**

The area under the curve (AUC) for the ACR 2011c and 2016c was 0.870 and 0.845, respectively, and the sensitivity and specificity were 78.0% and 96.0% for the 2011c and 70.5% and 98.5% for the 2016c, respectively. The FS scale and its subscales were confirmed to exhibit good internal consistency, and they were significantly correlated with the FIQR, thereby indicating adequate construct validity. Using a lower cutoff value 11 points for the FS scale score based on the generalized pain requirement might be a more effective approach in the Chinese population; this approach yielded an AUC of 0.923 and a sensitivity of 87.0% and specificity of 97.5%.

**Conclusion:**

The 2011c and 2016c are reliable instruments for diagnosing fibromyalgia patients in China. The FS scale could be a valid tool to assist in fibromyalgia diagnosis, and a cutoff value 11 points is more suitable in Chinese patients.

**Trial registration:**

ClinicalTrials.gov ID: NCT03381131

## KEY MASSAGES

The Chinese version of the ACR 2011c and 2016c are valid tools for fibromyalgia diagnosis; and a cutoff value 11 points for FS might be more suitable to assist in fibromyalgia diagnosis in Chinese population. The Chinese 2011c and 2016c for fibromyalgia diagnosis can be found as an appendix to this article.

## Introduction

Fibromyalgia is a chronic disorder characterized by generalized pain, fatigue, insomnia, and mental disorders, such as anxiety and depression [1,2]. This condition affects up to 5% of the general population globally, making it the second most common rheumatologic condition (trailing only osteoarthritis) [3]. However, in mainland China, it remains challenging to diagnose fibromyalgia, leading to a high frequency of delayed diagnoses and misdiagnoses. A recent cross-sectional study reported that 90% of fibromyalgia patients were misdiagnosed at their first visit complaining of fibromyalgia symptoms [4], and fibromyalgia was correctly diagnosed an average of 2 years later [5]. Moreover, the misdiagnosis rate of fibromyalgia is also high, reaching 87% [6].

There are several hypotheses regarding the difficulty of diagnosing fibromyalgia in China. First, there is a lack of knowledge and a misunderstanding of this syndrome among doctors, even rheumatologists [7]. Second, according to previous findings, Chinese patients may have milder fibromyalgia-related symptoms and a better quality of life (QOL) than individuals from other cultural and ethnic groups [4]; therefore, the ACR diagnostic criteria for fibromyalgia may not be generalizable to the Chinese population. Third, the diagnostic level of fibromyalgia in China needs to be improved.

Since fibromyalgia is a complex clinical syndrome without distinct biomarker(s) [8], the diagnosis of fibromyalgia was and still is mostly made by clinical presentation and excludes alternative diagnoses. To facilitate the diagnosis of fibromyalgia, in 1990, the ACR developed fibromyalgia classification criteria that require the presence of generalized pain for 3 months and physical examination to identify tenderness at 11 or more of the 18 specific tender point counts (TPCs) [9]. Although the ACR 1990 criteria (1990c) is recognized as the gold standard for fibromyalgia diagnosis, it is not widely used because of the challenging nature of TPC examination, which is the main reason for delay diagnoses of fibromyalgia [10]. In addition, the 1990c lacks the ability to characterize the clinical symptoms and severity and the ability to monitor the progression of the disease. Therefore, ACR developed new preliminary diagnostic criteria for fibromyalgia in 2010 [11] as well as a self-report questionnaire for patient surveys and clinical research in 2011 [12]; in these new criteria, the TPC examination was eliminated. In 2016, ACR published a revision to the 2010/2011 fibromyalgia diagnostic criteria [13] that validated their utility, made modifications to the previously identified problems, and addressed the clinical importance of the fibromyalgia severity (FS) scale. Although the above-mentioned criteria were developed and validated meticulously elsewhere in the world, these criteria were not evaluated and validated in China before being adopted as the diagnostic standard. This is problematic because symptoms and symptom severity are subjective and are likely influenced by racial and cultural backgrounds.

Therefore, to provide a solid foundation for clinical diagnosis, and future investigation and researches, this study aimed to verify the applicability, reliability and validity of the ACR 2011 criteria (2011c) and 2016 criteria (2016c) for fibromyalgia in China, and assumed that the 2011c and 2016c were reliable instruments for diagnosing Chinese patients with fibromyalgia. It also aimed to examine the clinical diagnostic perspective of the 2011c and 2016c for identifing the optimal cutoff score for using the FS scale to diagnose fibromyalgia among Chinese patients.

## Methods

### Sampling size estimation and research design

The Power Analysis and SampleSize (PASS) version 11.0 software (www.ncss.com) was used to calculate the sample size of this diagnostic test. A previous ACR 2016c assessment study conducted in a Norwegian fibromyalgia population resulted in a sensitivity of 88.8% and a positive ratio of 86% according to the ACR 1990c [14]. Based on the Confidence level (1-α) = 0.95 and Confidence interval width (two sided)= 0.2, a total of 178 patients with fibromyalgia should be investigated. Considering 10% questionnaires might be failed to fill qualifiedly, there should be about 200 cases in fibromyalgia group.

This multicentre diagnostic trial was conducted from 1 October 2018, to 30 September 2021. We recruited 200 patients with fibromyalgia who met the 1990c from the rheumatology clinic at 6 hospitals. The rheumatoid arthritis (RA) group was matched with fibromyalgia group based on gender and age. The study protocol was approved by the ethics committee of the Guang’anmen hospital (approval number 2018-059-KY) and have been performed in accordance with the Declaration of Helsinki, and written informed consent was obtained from all participants. Investigator training, including tenderness site examination, was conducted prior to the clinical study.

### Patient selection

The inclusion criterion for fibromyalgia participants was receiving a diagnosis of fibromyalgia based on the 1990c at the first outpatient visit regardless of whether they had a fibromyalgia diagnosis previously. The inclusion criteria for control participants were (i) no previous diagnosis of fibromyalgia and not meeting the criteria for fibromyalgia on the day of the visit and (ii) previously diagnosed with RA by a rheumatologist according to ACR 1987 criteria [15] or 2010 criteria of RA [16]. Patients with (i) severe mental diseases, including schizophrenia, personality disorders, dissociative disorders, mood disorders, post-traumatic stress disorder, etc.; (ii) and fractures, defined neuropathic causes or other nonrheumatic causes of pain were excluded from the study.

### Study procedure

Patients visiting the outpatient clinic for body pain were first screened for the diagnosis of fibromyalgia using the 1990c by a trained investigator. If they met the inclusion criteria, their demographic and disease-related information was collected after informed consent was obtained. The assessment of fibromyalgia based on the 2011c and 2016c was completed by the patients themselves and the same investigator separately at the first visit and the second visit within 7 to 14 days. The investigators in this study were well trained and experienced in checking the TPC and using the scale.

### Translation of Chinese versions of the 2011c and 2016c

With the permission of the original author by e-mail correspondence, the 2011c and 2016c were translated into Chinese following an adequate translation procedure according to optimal standards [17]. Two native Chinese-speaking experts, including one physician and one layperson, independently translated the 2011c and 2016c. Then, two native English-speaking experts, including one physician and one layperson, reverse translated the Chinese versions of the 2011c and 2016c reversely. After the translation, two bilingual multidisciplinary experts compared the original versions with the translated versions and modified and improved the translated versions to ensure that the translated versions were fully understandable, and the experts verified the cross-cultural semantic consistency between the two versions. Two bilingual patients volunteered to complete the translated versions and to discuss the translated items with their physicians to ensure that the semantics were clear; then, the translated versions were further modified and improved. Finally, the ACR 2011c and 2016c were translated and culturally adapted successfully.

### Questionnaires

The FS scale includes two subscales: the generalized pain index (WPI) and the symptom severity scale (SSS). The WPI assesses the number of areas in which the patient has had pain over the last week with a score ranging from 0 to 19. The SSS measures the severity level of the following symptoms over the previous week with a score ranging from 0 to 12: fatigue, waking unrefreshed, cognitive symptoms and the extent of somatic symptoms. A higher score on the SSS indicates more severe fibromyalgia-related symptoms. The sum of the WPI and SSS generates the FS scale score, which ranges from 0 to 31; higher scores indicate worse fibromyalgia severity [12,13].

The diagnosis of fibromyalgia can be made only if 1) the diffuse symptoms last for at least 3 months and 2) the symptoms are not better explained by other disorders. Furthermore, the 2011c for fibromyalgia were as follows: WPI ≥7 and SSS score ≥5 or WPI score from 3–6 and SSS score ≥9. The 2016c criteria for fibromyalgia were as follows: WPI ≥7 and SSS score ≥5 or WPI from 4 to 6 and SSS score ≥9. Additionally, there must be generalized pain involving at least 4 of the following 5 areas (excluding jaw, chest and abdomen pain).

The revised fibromyalgia impact questionnaire (FIQR) is a self-administered questionnaire with 21 questions across three subscales: (1) the ‘function’ subscale focuses on the ability to perform large muscle tasks, (2) the ‘overall impact’ subscale focuses on the overall impact of fibromyalgia, and (3) the ‘symptoms’ subscale focuses on the common fibromyalgia-related symptoms. The total FIQR score ranges from 0 to 100, with lower scores indicating more improvement or less negative impact, and this questionnaire has been validated in Chinese patients with fibromyalgia [18].

### Statistics

Statistical Package for Social Science (SPSS) version 26.0 (IBM SPSS, China) was used for statistical analysis. The ACR 2011c and 2016c for validity, reliability, and the relevant Consensus-based standards for the selection of health measurement instruments (COSMIN) checklist [19] were applicable for the analysis.

Qualitative data were represented as numbers and percentages, and continuous variables were summarized as mean and standard deviation (SD), as well as the median and interquartile range (IQR) if they were nonnormal distributed. The fibromyalgia group and RA group were compared with the Mann–Whitney U test or independent sample T-test for the continuous variables and the chi-square statistic for qualitative data. The standardized mean difference effect size statistical was used in the comparisons of the continuous variables between the fibromyalgia group and RA group.

The test-retest reliability of the WPI, SSS and FS scales was assessed with Spearman’s correlation coefficient, with 1 indicating a strong correlation and 0 indicating a weak correlation. Cronbach’s α coefficient was analysed to assess the reliability and internal consistency of the 2011c and 2016c, and the value was in the range of 0.7 to 0.9, representing good internal consistency [20]. Construct validity was analysed by assessing the FS scale, and its subscales (WPI and SSS score) were compared with the FIQR, including the FIQR total score and its subscales (FIQR-function, -overall, and -symptom scores) by Spearman correlation coefficient analysis.

The receiver operator characteristic (ROC) analyses of the 2011c and 2016c were generated based on whether patients met these two diagnostic criteria. The area under the curve (AUC), sensitivity and specificity were calculated to assess the reliability and validity of the 2011c and 2016c. In addition, positive and negative predictive values (PPV and NPV) and positive and negative likelihood ratios (PLR and NLR) were calculated to assess accuracy. ROC curves were analysed to determine whether new cutoff values of the FS scale combined with the ‘at least 4 of 5 regions’ criterion could lead to better diagnostic indices than the 2010c and 2016c.

All statistical tests were two-sided. To avoid comparison bias, p values less than 0.01 were considered statistically significant.

## Results

### Comparison of demographic and clinical characteristics

The demographic and clinical characteristics of the fibromyalgia group and RA group are shown in Table 1. The mean age was 48.7 in fibromyalgia group and 49.2 in RA group, and the proportion of females was 87%. A shorter symptom duration, higher WPI, SSS, and FS scale scores, as well as more TPC were observed in the fibromyalgia group (p< 0.001). The percentages of those who met the 2011c and 2016c were significantly greater in the fibromyalgia group than in the RA group (*p*< 0.001).

**Table 1.** Demographic and clinical characteristics between the fibromyalgia and rheumatoid arthritis groups (fibromyalgia diagnosis met the ACR 1990c).

	Fibromyalgia group (*n* = 200)	Rheumatoid arthritis group (*n* = 200)	*p* value	Effect Size
Females (*n* (%))	174 (87.0)	174 (87.0)	1.00	/
Age, years (mean (SD))	48.7 (13.4)	49.2 (13.2)	0.73	0.038
Marital status (*n* (%))			0.91	/
Unmarried	21 (10.5)	18 (9.0)		
Married	167 (83.5)	174 (87.0)		
Divorced	8 (4.0)	4 (2.0)		
Widowed	4 (2.0)	4 (2.0)		
Occupational status (*n* (%))^a^			0.86	/
Employed	88 (44.2)	82 (41.8)		
Retired	70 (35.2)	78 (39.8)		
Homemaker	21 (10.6)	19 (9.7)		
Unemployed	20 (10.1)	17 (8.7)		
Educational level (*n* (%))^b^			0.62	/
Did not receive formal education	5 (2.5)	1 (0.5)		
Completed <9^th^ grade	45 (22.6)	46 (23.1)		
Completed high school	48 (24.1)	56 (28.1)		
Completed college	80 (40.2)	84 (42.2)		
Received a postgraduate degree	21 (10.6)	12 (6.0)		
Symptom duration, months (median (IQR))	24.0 (6.0, 84.0)	49.5 (12.0, 144.0)	<0.001	0.31
TPC (mean (SD))	13.8 (2.5)	2.1 (2.6)	<0.001	−4.587
FS (mean (SD))	19.9 (5.8)	5.4 (4.0)	<0.001	−2.91
WPI	12.5 (4.4)	1.9 (2.3)	<0.001	−3.019
SSS	7.4 (2.7)	3.5 (2.5)	<0.001	−1.499
FIQR total score (median (IQR))	35.7 (24.9, 51.8)	15.3 (7.5, 28.2)	<0.001	−1.022
FIQR function score	18.0 (5.0, 35.0)	13.0 (4.0, 31.0)	0.39	−0.085
FIQR overall score	6.5 (2.0, 13.0)	0.0 (0.0, 1.0)	<0.001	−1.111
FIQR symptom score	50.0 (34.0, 61.0)	19.0 (11.0, 35.0)	<0.001	−1.349
ACR 2011c positive (n (%))	156 (78.0)	8 (4.0)	<0.001	/
ACR 2016c positive (n (%))	141 (70.5)	3 (1.5)	<0.001	/

*ACR*: American College of Rheumatology; 1990c: 1990 criteria; *SD: *standard deviation; *IQR*: interquartile range; *TPC*: tender point count; *FS*: fibromyalgia severity; *WPI*: widespread pain index; *SSS*: symptom severity scale; *FIQR*: revised fibromyalgia impact questionnaire; *2011c*, 2011 criteria; *2016c*, 2016 criteria.

^a^there was 1 fibromyalgia patient missing and 4 rheumatoid arthritis patients missing.

^b^there was 1 fibromyalgia patient and 1 rheumatoid arthritis patient missing.

/: inapplicability.

### The internal consistency reliability

The internal consistency and test-retest reliability were determined by examining the data of the first visit and the second visit in the fibromyalgia group. The results of Spearman’s correlation analysis for the FS scale, its subscales, and all 25 single items revealed highly positive correlations (ranging from 0.53 to 0.82) for each item in the two diagnostic criteria. Cronbach’s α coefficient was 0.82 for the total FS scale score, 0.84 for the WPI, and 0.65 for the SSS.

### The construct validity

The Spearman’s correlation coefficients between the FS scale and its subscales (WPI and SSS) and between the FIQR total score and its subscales were calculated to examine construct validity, and the results are shown in Table 2. The FS scale, WPI and SSS were significantly correlated with all the FIQR scores (total, function, overall, and symptom scores), which indicates good construct validity.

**Table 2.** FS construct validity: Spearman’s correlation coefficients among subscales of the FIQR and FS scale.

	FIQR total score	FIQR function score	FIQR overall score	FIQR symptom score
FS	0.487*	0.322*	0.401*	0.498*
WPI	0.292*	0.256*	0.307*	0.232*
SSS	0.589*	0.330*	0.398*	0.674*

*FS*: fibromyalgia severity, the sum of the WPI and SSS scores; *FIQR*: revised fibromyalgia impact questionnaire; *WPI*: widespread pain index; *SSS*: symptom severity scale.

* Correlation is significant at the 0.01 level (2-tailed).

### Validity analysis of ACR 2011c and 2016c

The AUC, sensitivity, specificity, PPV, NPV, PLR and NLR of the 2011c and 2016c are shown in Table 3. The high specificity of the 2011c (96.0%) and the 2016c (98.5%) indicates that those two criteria are reliable for diagnosing fibromyalgia patients in China; however, the sensitivity values were low (78.0% and 70.5% for the 2011c and 2016c, respectively) and the AUC values were 0.870 for the 2011c and 0.845 for the 2016c.

**Table 3.** Validity analysis of the ACR 2011c and 2016c, with the ACR 1990c serving as the gold standard.

Criteria	AUC (CI)	Sensitivity (95%CI)	Specificity (95% CI)	PPV (95% CI)	NPV (95% CI)	PLR (95% CI)	NLR (95% CI)
ACR 2011c	0.870 (0.83 2–0.908)	78.0 (71.5–83.4)	96.0 (92.0–98.1)	95.1 (90.3–97.7)	81.4 (75.7–86.0)	19.5 (9.9–38.4)	0.2 (0.2–0.3)
ACR 2016c	0.845 (0.804–0.886)	70.5 (63.6–76.6)	98.5 (95.3–99.6)	97.9 (93.6–99.5)	77.0 (71.2–81.9)	47.0 (15.3–144.0)	0.3 (0.2–0.4)

*ACR*: American College of Rheumatology; *2011c*, 2011 criteria; *2016c*, 2016 criteria; *1990c*, 1990 criteria; *AUC:* area under curve; *CI*: confidence interval; *PPV*: positive predictive value; *NPV*: negative predictive value; *PLR:* positive likelihood ratio; *NLR*: negative likelihood ratio. *FS*: fibromyalgia severity; *WPI*: widespread pain index; *SSS*: symptom severity scale; the 2011c include (1) a WPI ≥7 and a SSS score ≥5 or a WPI 3-6 and a SSS score ≥9, (2) symptoms present at a similar level of severity for at least 3 months; the 2016c include (1) a WPI ≥7 and a SSS score ≥5 or a WPI 4-6 and a SSS score ≥9, (2) widespread pain involving at least 4 of 5 specific areas (excluding the jaw, chest and abdomen), (3) diffuse symptoms lasting for at least 3 months.

### Characteristics comparison of patients positive or negative against 2011c and 2016c

Comparative analyses were performed to investigate the differences in characteristics and who were positive or negative based on the 2011c and 2016c among patients who met the 1990c, and the results are shown in Table 4. No differences were found in terms of TPC among 2011c positive, 2011c negative (which means also failed to satisfy 2016c), 2016c positive (which means also satisfied the 2011c), 2016c negative, and 2011c positive but 2016c negative groups. All inconsistencies occurred in the 15 2011c positive cases that failed to meet the 2016c, because all 15 cases did not meet the generalized pain requirement of 4 pain regions, and 2 cases did not have a WPI minimum score of 4 (the minimum scores were 3 in the 2011c).

**Table 4.** Differences between fibromyalgia patients according to their diagnostic condition, with the fibromyalgia diagnoses meeting the ACR 1990c.

	2011c positive (*n* = 156)	2011c negative (*n* = 44)	2016c positive (*n* = 141)	2016c negative (*n* = 59)	2011c positive & 2016c negative (*n* = 15)
Age, years (mean (SD))	48.4 (13.5)	49.9 (13.2)	48.5 (13.6)	49.1 (13.1)	46.7 (13.1)
Females (n (%))	138 (88.5)	36 (81.8)	125 (88.7)	49 (83.1)	13 (86.7)
Symptom duration, months (median (IQR))	36.0 (8.3, 96.0)	9.0 (5.0, 24.0)	36.0 (8.0, 96.0)	12.0 (5.0, 36.0)	24.0 (11.0, 60.0)
TPC (mean (SD))	13.8 (2.5)	14.1 (2.5)	13.8 (2.4)	13.8 (2.5)	13.1 (2.2)
FS (mean (SD))	21.4 (5.2)	14.5 (4.3)	15.0 (4.1)	21.9 (5.2)	16.6 (2.8)
WPI	12.9 (4.1)	10.7 (5.1)	10.1 (4.6)	13.4 (4.0)	8.3 (2.1)
SSS	8.4 (1.9)	3.7 (1.4)	4.9 (2.5)	8.5 (2.0)	9.3 (1.3)

*ACR*: American College of Rheumatology; *1990c,* 1990 criteria; *2011c*, 2011 criteria; *2016c*, 2016 criteria; *SD*: standard deviation; *IQR*: interquartile range; *TPC:* tender point count; *FS*: fibromyalgia severity; *WPI:* widespread pain index; *SSS*: symptom severity scale.

However, it seems that 1) males are less likely to meet either the 2011c or the 2016c, 2) the shorter the duration of FM is, the less likely it is for patients to meet either the 2011c or the 2016c, and 3) young patients tend to meet the 2011c but not the 2016c. Additionally, the patients who either satisfied the 2011c or 2016c had a longer symptom duration and higher WPI, SSS and FS scale scores than those who failed to meet the criteria.

### ROC analyses for the cutoff point of the FS scale based on the generalized pain criterion

Considering that 2016c is the latest set of ACR fibromyalgia criteria and the advanced standard that additionally applies the generalized pain criterion, we performed ROC analysis according to the cutoff score for the FS scale, which is based on the generalized pain criterion. As shown in Table 5, the results show higher AUC and sensitivity values but lower specificity values than the ACR original 2016c. The highest AUC value was 0.923 when the cutoff score of the FS scale was 11; at this score, the sensitivity and specificity were 87.0% and 97.5%, respectively.

**Table 5.** Receiver operator characteristic analyses based on the FS cutoff point in fibromyalgia patients meeting the generalized pain requirement of 4 pain regions.

FS cutoff point	AUC	Sensitivity	Specificity	PPV	NPV	PLR	NLR
FS ≥ 14	0.900	81.0	99.0	98.8	83.9	81	0.2
FS ≥ 13	0.918	84.5	99.0	98.8	86.5	84.5	0.2
FS ≥ 12	0.915	85.5	97.5	97.2	87.1	34.2	0.2
FS ≥ 11	0.923	87.0	97.5	97.2	88.2	34.8	0.1
FS ≥ 10	0.920	88.0	96.0	95.7	88.9	22.0	0.1
FS ≥ 9	0.913	88.5	94.5	94.1	89.2	16.1	0.1

*FS*: fibromyalgia severity; *AUC*: area under curve; *PPV*: positive predictive value; *NPV*: negative predictive value; *PLR*: positive likelihood ratio; *NLR*: negative likelihood ratio.

## Discussion

Our findings reveal that the 2011c and 2016c are valid instruments with high specificity that can be used to diagnose fibromyalgia in the Chinese population. The demographic characteristics and clinical features of the participants in this multicentre study were similar to those in a previous report of Chinese patients with fibromyalgia [4]. Our results also suggested that based on chronic pain (lasting for at least 3 months), a FS scale score ≥ 11 combined with generalized pain (pain involving at least 4 of the 5 areas) may be more suitable for use as modified 2016c and are worthy of further study to determine whether they could be used as valid tools for diagnosis in China.

Some adjustments had to be made when validating the 2011c and 2016c for Chinese fibromyalgia patients from the original versions of Wolfe et al. On the one hand, based on the advanced diagnostic concept of the 2016c that clarity was unnecessary with respect to fibromyalgia diagnosis in the presence of other diseases, we also eliminated this condition when using the 2011c for diagnosis. On the other hand, all items of the WPI and SSS are put into tables, and the interpretation of scoring criteria are marked as notes. These changes make the FS scale, WPI and SSS scoring procedures simpler and more comfortable for both patients and physicians. Thus, these changes result in a more rapid clinical diagnosis for fibromyalgia.

The results of the current study reveal that the 2011c and 2016c had strong reliability and a good level of internal consistency in China. These findings are acceptable and comparable with previous reports. The 2016c validation study in Korean patients with fibromyalgia also showed an acceptable Cronbach’s α coefficient of 0.942 (95% CI: 0.930–0.964) [21], similar to a previous French study [22]. However, a weak internal correlation of each item on the SSS was found; this was indicated by the Cronbach α coefficient of 0.65 for the SSS when considering both the 2011c and 2016c. Nevertheless, this relatively low reliability of the SSS scale was also shown in a few studies [23,24], which indicated the variance in the severity of fibromyalgia symptoms.

The 2011c and 2016c presented acceptable sensitivity (78.0% and 70.5%, respectively) and specificity values (96.0% and 98.5%, respectively) in Chinese patients with fibromyalgia. Those findings show that the 2011c tends to have lower sensitivity but higher specificity than criteria reported in validation studies from other countries, such as America (sensitivity: 86% and specificity: 90%)[13], Spain (sensitivity: 88.3% and specificity: 91.8%) [25], Italy (sensitivity: 79.8% and specificity: 91.7%) [26] and Norway (sensitivity: 93.9% and specificity: 71.3%)[14]. In contrast, a Japanese version that was tested in 462 fibromyalgia patients with 231 RA or osteoarthritis controls yielded a sensitivity of 64% and a specificity of 96% [24]. Similarly, the 2016c is likely to have lower sensitivity but higher specificity than those found in validation studies from America (sensitivity: 85% and specificity: 90%) [13], Italy (sensitivity: 78% and specificity: 90.5%) [26], Norwegian (sensitivity: 88.8% and specificity: 77.5%)[14] and South Korea (sensitivity: 93.1% and specificity: 90.7%) [21]. However, our findings are in line with a Spanish study that found a low sensitivity (75.6%) and a high specificity (99.7%) when patients were diagnosed both by 1990c and ACR 2011c [25]. Contrary to our findings, a Norwegian study found high sensitivity when using the Norwegian version of the 2011c (93.9%) and 2016c (88.8%) but low specificity values (71.3% and 77.5%, respectively) among patients who fulfilled the ACR 1990c [14]. Additionally, the AUC values for the 2011c and 2016c are 0.870 and 0.845, respectively, which is similar to the AUC value of 0.86 observed for the 2016c in the Norwegian study [14], higher than the AUC of 0.79 for the 2010c observed in a Spanish study[27] and lower than the AUC of 0.97 for the 2016c observed in a Korean study[21]. The sociocultural characteristics may explain the different results in terms of sensitivity and specificity among different populations. The social variables vary in fibromyalgia patients from different countries or races, such as culture [28], social factors [29], diet structure [30], body composition [31], anxiety and depression level [32,33], and past experiences [34] can interfere the clinical presentation and symptoms severity of fibromyalgia. Thus, the sociocultural factors should be considered as important factors to take into account in the diagnosis of fibromyalgia.

Among the 200 patients who satisfied the 1990c criteria, there were 156 patients (78.0%) who met the 2011c and 141 patients (70.5%) who met the 2016c criteria. This resulted in agreement in 141 cases (70.5%) and disagreement in 15 cases (7.5%). This result came about because 15 of the 2011-positive cases failed to meet the new generalized pain requirement, and 2 cases also failed to meet the WPI minimum score of 4. The 2016c does not misclassify positive patients who were negative based on the 2011c. All the changes in criteria status occurred in those who were positive based on the 2011c, amounting to almost 10% (15/156) of all 2011c-positive subjects. The 2016c is revised from 2011c to ensure that patients with regional pain syndromes would not be misclassified as having fibromyalgia. However, the cost of this assurance was that nearly 30% (59/200 cases) of patients diagnosed by the 1990c and 10% (15/156 cases) of patients diagnosed by the 2011c fail to meet the 2016c. Thus, these changes were useful but costly because they reduced the sensitivity of the diagnostic criterion and enhanced the limitations in reliability and validity.

It seems that an improvement in sensitivity without a significant decrease in specificity can be achieved by using a lower cutoff score for the FS scale instead of both of its subscales (WPI and SSS) among Chinese patients. Score on the FS scale and its subscales were significantly different between the fibromyalgia and nonfibromyalgia control groups. The FS scale could differentiate fibromyalgia from other chronic pain disorders with acceptable sensitivity, specificity, PPV and NPV values. Using a lower cutoff score for the FS scale (≥11) based on meeting the generalized pain might be a more effective approach in the Chinese population, yielding a higher AUC of 0.923 and a sensitivity and specificity of 87.0% and 97.5%, respectively. This finding indicates that symptoms among Chinese patients with fibromyalgia are likely to be less severe, which is consistent with our previous study [4]. The use of a lower cutoff score for the FS scale should be researched further to test its reliability in assessing the symptom intensity and fibromyalgia diagnosis for Chinese patients.

There are some limitations in the current study. Firstly, the disease duration of patients in the fibromyalgia group was similar to that in our previous report (median duration of fibromyalgia: 24.0 vs. 24.0 months) [4] but was shorter than that in the RA group (median: 24.0 vs. 49.5 months). Additional Chinese epidemiological studies are needed to determine whether the onset age of fibromyalgia is older than that of RA. Secondly, none of the fibromyalgia subjects were recruited from a primary care setting; thus, they probably do not reflect the performance of these criteria in the general population. Thirdly, the study solely matched the control group of RA patients may limit the generalizability of the findings. However, the feasibility of clinical implementation if involving myofascial pain syndrome and generalized osteoarthritis as control group is challenged because of their low prevalence, so the study did not include other diseases as control.

Although the ACR 2011c and 2016c were validated at the tertiary level of care, the present study shows acceptable validity of those tools for fibromyalgia diagnosis for application in research settings among Chinese patients with fibromyalgia in a fairly large sample size. It remains to be clarified whether the new cutoff value for the FS scale could yield better AUC, sensitivity, and specificity values for the diagnosis of fibromyalgia among Chinese patients. This approach should be examined further in clinical and research settings to enhance the treatment response and outcomes of this disorder.

In conclusion, the 2011c and 2016c are reliable instruments for diagnosing fibromyalgia patients in China. The FS scale could be a valid tool to assist in fibromyalgia diagnosis, and a cutoff value 11 points is more suitable in Chinese patients.

## Data Availability

The data that support the findings of this study are available from the Ethical Committees of Guang ‘anmen Hospital and the corresponding author on reasonable request.
